# Antioxidant, Anti-Inflammatory, and Cytotoxic Activity of Extracts from Some Commercial Apple Cultivars in Two Colorectal and Glioblastoma Human Cell Lines

**DOI:** 10.3390/antiox10071098

**Published:** 2021-07-08

**Authors:** Aurita Butkeviciute, Vilma Petrikaite, Vidmante Jurgaityte, Mindaugas Liaudanskas, Valdimaras Janulis

**Affiliations:** 1 Department of Pharmacognosy, Lithuanian University of Health Sciences, Sukileliu av. 13, LT-50162 Kaunas, Lithuania; Mindaugas.Liaudanskas@lsmu.lt (M.L.); Valdimaras.Janulis@lsmuni.lt (V.J.); 2 Laboratory of Drug Targets Histopathology, Institute of Cardiology, Lithuanian University of Health Sciences, Sukileliu av. 13, LT-50162 Kaunas, Lithuania; Vilma.Petrikaite@lsmuni.lt (V.P.); vidmante.jurgaityte.19@ucl.ac.uk (V.J.); 3 Institute of Physiology and Pharmacology, Lithuanian University of Health Sciences, A. Mickeviciaus 9, LT-44307 Kaunas, Lithuania

**Keywords:** apple, hyaluronic acid, hyaluronidase, HT-29, U-87, DPPH, ABTS, CUPRAC, FRAP

## Abstract

Cancer initiation and development are closely related to oxidative stress and chronic inflammation. The aim of this study was to evaluate apple extracts and individual tritepenes antioxidant, anti-inflammatory, and cytotoxic activities. Dry extracts of apple were analyzed by HPLC-PDA. A hyaluronidase inhibition assay was selected to determine the anti-inflammatory effect. Cytotoxic activities against human colon adenocarcinoma cell line (HT-29) and human glioblastoma cell line (U-87) were determined using MTT, cell colony formation, and spheroid growth assays. Radical scavenging and reducing activities were evaluated using DPPH, ABTS, FRAP, and CUPRAC assays, respectively. The apple extracts inhibited hyaluronidase from 26.38 ± 4.4% to 35.05 ± 3.8%. The AAW extract possessed the strongest cytotoxic activity (EC_50_ varied from 113.3 ± 11.11 µg/mL to 119.7 ± 4.0 µg/mL). The AEW extract had four and five times stronger antiradical activity when determined by ABTS and DPPH, and two and eight times stronger reducing activity when evaluated by CUPRAC and FRAP, respectively. Understanding the mechanisms of apple extracts and individual triterpenes as hyaluronidase inhibitors and antioxidants related in cancer development may be a benefit to future study in vivo, as well as cancer prognosis or the development of new, innovative food supplements, which could be used for chronic disease prevention.

## 1. Introduction

Apples are one of the most consumed fruits worldwide [1]. In nutrition, apples are a relevant source of bioactive compounds [2]. They are rich with phenolic [3] and triterpene [4] compounds, vitamins [5], micro- and macroelements [6], organic acids [7], and fiber [3]. Phenolic compounds determined in apple fruits act on human biological systems. They possess anti-inflammatory and antioxidant activities, neutralizing harmful reactive free radicals that trigger structural damage to the body’s macromolecules, which, in turn, is directly related to the progression of various diseases including inflammatory diseases and cancer [8,9]. The pentacyclic triterpenes determined in apples are characterized by various pharmacological activities (i.e., they decrease the concentration of low-density lipoproteins and glucose, and inhibit atherosclerotic plaque formation and inflammation [10,11,12]. A wide array of research has disclosed the potential for many antioxidant and anti-inflammatory bioactive compounds to prevent carcinogenesis.

Over the past decade, extensive scientific progress has been made in understanding carcinogenic processes at the cellular and molecular level. This has led to an encouraging viewpoint on cancer prevention, termed “chemoprevention”, the purpose of which is to suppress or reverse the development and progression of precancerous cells through the use of non-cytotoxic agents or phytochemicals [13]. Previous studies have revealed that cancer initiation and development are closely related to oxidative stress and chronic inflammation [14]. Flare-ups of oxidative stress and chronic inflammation can influence cellular genetic instability and epigenetic modifications, leading to the deregulation of cancer suppressors and oncogenes that promote the initiation of carcinogenesis [15]. It has been established that many elements of a vegetarian diet, especially fruits and vegetables, comprise various bioactive compounds that can protect cells or tissues from harmful oxidative and inflammation-mediated tissue damage, and as a consequence can prevent the development of cancer. Previous research has been performed to investigate cytotoxic biological active compounds from turmeric, broccoli, green tea, grapes, berries, pomegranate, and apples. Between those dietary sources, apples have been determined to be a source of many antioxidants and anti-inflammatory agents [14]. For this reason, it is important to determine the effects of apple extracts on cell viability, and to carry out more-detailed studies of their mechanisms of action.

Colon cancer is the third most widespread cause of cancer death in the world, due to the long precancerous stage of colorectal cancer [16]. Meanwhile, glioblastoma is the deadliest form of brain cancer, accounting for more than 50% of primary brain tumors [17,18]. The tumor microenvironment occupies an important role in controlling the destiny of cancer cells and strongly dependent on the composition of the extracellular matrix [19,20,21]. One of the most relevant enzymes in inflammatory response is hyaluronidases, which depolymerize hyaluronic acid (the main constituent of the extracellular matrix), which modulates the activity of many pathological processes [22,23]. Hartheimer et al. showed that hyaluronic acid deposition is upregulated in most malignancies. In human cancers in particular, hyaluronic acid content is usually higher in cancerous than in normal tissues [17]. The scientific literature describes that colon cancer and glioblastoma, among other kinds of cancer, are considered enriched with hyaluronic acid [18,24]. In that type of tumor, hyaluronic acid may keep up tumor growth by stimulating anchorage-independent growth and the proliferation of tumor cells [24]. Due to a wide variety of signaling pathways and pathological processes regulated by hyaluronidase [13,25], these tumors are very attractive targets in the study of new plant-based bioactive components that could potentially inhibit hyaluronidase and, as a result, inflammation processes, thereby maintaining the homeostasis of hyaluronic acid.

A hyaluronidase inhibition assay was selected to establish the anti-inflammatory activity of whole-apple and apple peel samples along with standard substances of the predominant triterpenes of apple samples. To thoroughly determine the antioxidant effects of apple extracts, four different antioxidant capacity assays, namely, DPPH and ABTS free radical scavenging and copper (CUPRAC) and ferric (FRAP) reducing activities in vitro were selected. Consequently, two human cell lines of different origins, namely, human colon adenocarcinoma (HT-29) and human glioblastoma cell line (U-87), were selected to evaluate the cytotoxic activities of apple extracts and enriched phenolic and triterpene compounds. As far as we know, no research has previously compared cytotoxic activities with colorectal and glioblastoma human cell lines, anti-hyaluronidase and free radical scavengers, the ferric and copper reducing activities of apple extracts, and predominant triterpenes. The evaluation of apple extracts with potentially active anti-hyaluronidase, antioxidant, and chemoprevention substances could be further functionalized in food supplements, functional foods, or value-added ingredients, or integrated into phytotherapy practices.

The aim of this study was to evaluate the qualitative and quantitative variabilities of triterpenes and phenols in whole apple and apple peels extracts, and to estimate their antioxidant, anti-inflammatory, and cytotoxic activities.

## 2. Materials and Methods

### 2.1. Plant Materials

This study included the apple cultivars Ligol, Rubin, and Auksis, which were grown in industrial gardens, as well as Kostele and Paprastasis antaninis, recorded in the List of the National Plant Genetic Resources. The apple trees were grown in the experimental orchard of the Institute of Horticulture, Lithuanian Research Centre for Agriculture and Forestry, Babtai, Lithuania (55°60′ N, 23°48′ E). The apples harvested in September 2020 were immediately lyophilized and applied to the research.

### 2.2. Cell Cultures

Human colon adenocarcinoma cell line (HT-29) and human glioblastoma cell line (U-87) were obtained from the American Type Culture Collection (ATCC, Manassas, VA, USA). Human fibroblasts were provided by prof. Helder A. Santos (University of Helsinki, Finland). Cancer cell lines were cultured in DMEM Glutamax medium supplemented with 10% FBS and 1% antibiotics at 37 °C with 5% CO_2_ in a humidified atmosphere. Cell cultures were grown to 70% confluency, trypsinized with 0.125% TrypLE Express solution before passage, and used until passage 20.

### 2.3. Chemicals and Solvents

All solvents, reagents, and standards used were of analytical grade. Standards used in HPLC analysis: ursolic acid, oleanolic acid, betulinic acid, corosolic acid, reynoutrin, (+)-catechin and (−)-epicatechin were obtained from Sigma-Aldrich GmbH (Buchs, Switzerland). Hyperoside, rutin, quercitrin, phloridzin, procyanidin B1, procyanidin B2, and chlorogenic acid standards purchased from Extrasynthese (Genay, France). Avicularin, procyanidin C1, and isoquercitrin were purchased from Chromadex (Santa Ana, CA, USA).

Chemicals applied in anti-inflammatory activities assay: 300–1000 U/mg hyaluronidase (HYAL) from bovine testes type VIII lyophilized powder, hyaluronic acid (HA) sodium salt from *Streptoccocus equi* bacterial glycosaminoglycan polysaccharide, disodium tetraborate decahydrate (Na_2_B_4_O_7_ × 10H_2_O), 36.5–38% hydrochloric acid, ≥98% sodium formate, and ≥99.5% sodium chloride acquired from Sigma-Aldrich GmbH (Buchs, Switzerland). Sodium chloride solution 0.9% was obtained from B. Braun Melsunger AG (Melsungen, Germany). Bovine serum albumin (BSA) pH 7 lyophilized from serum was obtained from Biowest SAS (Nuaille, France) and 98% 4-dimethylaminobenzaldehyde (DMAB) was acquired from AlfaAesar TermoFisher GmbH (Kandel, Germany).

Reagents used in antioxidant activities assay: 2,2′-azino-bis (3-ethylbenzothiazoline-6-sulfonic acid (ABTS), 6-hydroxy-2,5,7,8-tetramethylchroman-2-carboxylic acid (Trolox), potassium persulfate (K_2_S_2_O_8_), copper (II) chloride (CuCl_2_), ammonium acetate, neocuproine, 2,2-diphenyl-1-picrylhydrazyl (DPPH), and sodium acetate acquired from Scharlau (Barcelona, Spain). Iron (III) chloride hexahydrate (FeCl_3_ × 6H_2_O) was obtained from Vaseline-Fabrik Rhenania (Bonn, Germany), and 2,4,6-tri(2-pyridyl)-s-triazine (TPTZ) was obtained from Carl Roth (Karlsruhe, Germany).

Chemicals used in cytotoxic activities assay: ≥97% 3-(4,5-dimethylthiazol-2-yl)-2,5-diphenyl tetrazolium bromide (MTT) and ≥90% crystal violet purchased from GmbH (Buchs, Switzerland), 16% paraformaldehyde was obtained from Thermo Scientific (Thermo Fisher Scientific Inc., Waltham, MA, USA). The plasticware for cell cultures was obtained from Techno Plastic Products (Trasadingen, Switzerland), Corning (Corning, NY, USA), and Thermo Fisher Scientific (Waltham, MA, USA). Dulbecco’s modified Eagle high glucose medium (DMEM Glutamax), TrypLETM Express reagent, fetal bovine serum (FBS), penicillin/streptomycin solution (100×), and phosphate-buffered saline (PBS) were purchased from Gibco (Thermo Fisher Scientific Inc., Waltham, MA, USA). NanoShuttle magnetic nanoparticles were obtained from Nano3D Biosciences Inc. (Houston, TX, USA).

Solvents used in the study: 99.9% acetonitrile, 99.8–100.5% acetic acid, 99.9% acetone acquired from Sigma-Aldrich GmbH (Buchs, Switzerland); 96% ethanol was obtained from Stumbras AB (Kaunas, Lithuania) and 99.5% dimethyl sulfoxide (DMSO) was obtained from Scharlab S.L. (Barcelona, Spain). In this research, we applied deionized water prepared by a Milli-Q (Millipore, Bedford, MA, USA) water purification system.

### 2.4. Preparation of Apple Samples

Samples of whole apple and apple peel were prepared as described by Butkeviciute et al. [26].

### 2.5. Preparation of Dry Phenolic and Triterpene Extracts

During the assay of phenols, 20 g of lyophilized whole apple powder (exact weight) was weighed, added to 100 mL of 70% (*v/v*) ethanol, and extracted in a Sonorex Digital 10 P ultrasonic bath (Bandelin Electronic GmbH & Co. KG, Berlin, Germany) at room temperature for 20 min. The received extract was filtered through a paper filter, and the residue on the filter was washed with 70% (*v/v*) ethanol in a 100 mL flask until the accurate volume was achieved. During the assay of triterpenes, 20 g of lyophilized whole apple and apple peel powder (exact weight) was weighed, added to 100 mL of 99.9% (*v/v*) acetone, and extracted in the same ultrasonic bath at room temperature for 10 min. The prepared extract was filtered through a paper filter, and the residue on the filter was washed with acetone in a 100 mL flask until the accurate volume was achieved. The extraction was repeated three times, until the lyophilized apple and apple peel powder were completely exhausted.

Prepared extracts of apple samples were dried in an IKA RV 10 rotary evaporator (Staufen, Germany) at 40 °C until the acetone and ethanol were evaporated. Dry residues were desiccated in a vacuum drying oven Binder VD (BINDER GmbH, Planegg, Tuttlingen, Germany) to accomplish the evaporation of water and volatile compounds (temperature: 70 °C; variation in weight between counting: until 0.01 g) and counting the difference in raw lyophilized substance weight before and after drying. Data were recalculated for the absolute dry lyophilizate weight. The obtained dry whole apple and apple peel extracts were held in dark glass vessels (abbreviations shown in Table 1).

### 2.6. Determination of Phenolic Compounds by HPLC-PDA Method

Qualitative and quantitative HPLC analyses of phenolic compounds from the whole apple extracts were determined by applying the method described by Liaudanskas et al. [27].

### 2.7. Determination of Triterpene Compounds by HPLC-PDA Method

The triterpenes of the whole apple and apple peel samples were separated, identified, and quantified by applying HPLC similarly to the technique described in previous studies by Butkeviciute et al. [26].

### 2.8. Antioxidant Activity Assays

Antioxidant activities of the dry apple and apple peel extracts were determined by four different in vitro spectrophotometrical assays using a spectrophotometer (Spectronic CamSpec M550, Garforth, UK). For antioxidant activity assessment, all dry apple and apple peel extracts were dissolved in 70% ethanol until full dissolution, achieving a concentration of 30 mg/mL.

To perform the DPPH^•^ free radical scavenging assay, 3 mL of DPPH^•^ solution was mixed with 10 μL apple and apple peel extracts. A decrease in absorbance was measured after 30 min at λ = 517 nm [28]. To perform the ABTS^•+^ free radical scavenging assay, 3 mL of ABTS^•+^ solution was mixed with 10 μL apple and apple peel extracts. A decrease in absorbance was measured after 30 min at λ = 734 nm [28]. The FRAP solution included TPTZ (0.01 M dissolved in 0.04 M HCl), FeCl_3_ × 6H_2_O (0.02 M in water), and an acetate buffer (0.3 M, pH 3.6) (ratio 1:1:10). During the analysis, 3 mL of a freshly produced FRAP reagent was mixed with 10 μL apple and apple peel extracts. An increase in absorbance was estimated at λ = 593 nm [29]. The CUPRAC solution included copper (II) chloride (0.01 M in water), an ammonium acetate buffer solution (0.001 M, pH = 7), and neocuproine (0.0075 M in ethanol) (ratio 1:1:1). During the analysis, 3 mL of CUPRAC reagent were mixed with 10 μL apple and apple peel extracts. An increase in absorbance was evaluated at λ = 450 nm [30].

The evaluation of the free radical scavenging and reductive activities of apple extracts was counted using a Trolox calibration curve, which calculates a μmol Trolox equivalent (TE) per gram of the absolute dry weight (DW). The TE was calculated according to the formula TE = c × V/m (μmol/g), where c is the concentration of Trolox determined from the calibration curve (in μM), V is the volume of the apple extract (in L), and m is the weight (precise) of the lyophilized apple powder (in g).

### 2.9. Anti-Inflammatory Activities Assays

Hyaluronidase inhibition was evaluated by establishing the concentration of N-acetylglucosamine eliminated from sodium hyaluronate [31]. In the first stage of the HYAL inhibition activities assay, the needed reagents were prepared. A buffer consisting of 0.2 M sodium formate, 0.1 M NaCl, and 0.2 mg/mL BSA was prepared, with a pH value supported to 3.68 with formic acid. HA sodium salt was dissolved in purified water (5 mg/mL) and stored at 4 °C. HYAL lyophilized powder was dissolved in 0.9% NaCl solution (900 U/mL). Disodium tetraborate powder was dissolved in purified water (0.8 M) at 90–100 °C in a dry block Biosan TDB-12 thermostat (Biosan SIA RoHS, Riga, Latvia) until completely dissolved. DMAB was evaluated by dissolving 2 g DMAB in a mixture of 2.5 mL of 10 N HCl and 17.5 mL glacial acetic acid, then further diluting 1:1 with glacial acetic acid and using without delay. To estimate HYAL inhibition, all dry whole apple and apple peel extracts were dissolved in 1 mL 99.5% DMSO with full dissolution, obtaining a concentration of 30 mg/mL.

To carry out the HYAL inhibition activities assay, the following were added to 2.5 mL volume Eppendorf tubes: 100 μL H_2_O, 25 μL buffer pH 3.68, and a 25 μL mixture of 5 mg/mL HA in 25 μL buffer pH 3.68 (ratio 1:1). Next, 25 μL of DMSO was added into positive and negative control groups, and 25 μL of whole apple and apple peel extracts were added into the remaining tubes; 25 μL 900 U/mL HYAL solution was added into well-mixed test samples, except for the negative control group. All reagents were mixed and incubated at 37 °C for 45 min in a HERAcell 150i CO_2_ incubator (Thermo Fisher Scientific Inc., Waltham, MA, USA). After incubation, 30 μL of 0.8 M disodium tetraborate was added and heated at 100 °C for 5 min. Tested samples were cooled at room temperature, then 375 μL of DMAB solution was added (diluted) and incubated at 37 °C for 30 min. Prepared solutions were transferred into 96-well microtiter plates (200 μL/well). The absorbances of the test solutions were measured at 584 nm using a Tecan Spark 20 M multiscan plate reader (Tecan Trading AG, Mannedorf, Switzerland). HYAL inhibition (%) was calculated according to the following formula: HYAL inhibition (%) = 100 × (1 – [Absorption of sample/Absorption of control]). The assay was done in triplicate.

### 2.10. Cell Viability Assay

The activity of whole apple and apple peel extracts and the standard substances of triterpenes were analyzed for human colorectal carcinoma (HT-29) and glioblastoma (U-87) cell viability using an MTT assay as described by Grigalius and Petrikaite [32]. Hill fit to whole apple and apple peel extracts and standard substance dose–cell metabolic activity (absorbance) curves were applied and the results of the cell viability assay were expressed as effective concentration (EC_50_) values, reducing cell viability by 50%.

### 2.11. Cell Colony Formation

The activity of ursolic, oleanolic, betulinic and corosolic acids on the clonogenicity of cancer cells was studied by assessing their effects on the colony-forming capabilities of cells. HT-29 cells were seeded in 6-well plates in a volume of 2 mL (250 cells/well) and treated with 15 μL of individual triterpene compounds solutions. The concentrations of the triterpene compounds were used according to their EC_50_ values calculated from the MTT assay. Cells were incubated in a humidified atmosphere containing 5% CO_2_ at 37 °C. After 10 days, the cells were washed with PBS and fixed with 4% paraformaldehyde solution in PBS for 15 min. The cells were then washed with PBS two more times, incubated with 0.1% aqueous crystal violet solution for 20 min, and washed with sterile deionized water. Pictures were taken using the G:BOX gel documentation system (Syngene International Ltd., Bengaluru, India) and Genesys software (Syngene International Ltd.). The number and percentage areas of colonies were calculated.

### 2.12. Spheroid Growth Assay

Spheroids were formed using a magnetic 3D bioprinting method. The cancer cells were mixed with human fibroblasts (at a 1:1 ratio) to better depict the tumor microenvironment. The cancer cells were then incubated with 20 μL nanoparticles for 8–10 h and resuspended and seeded into ultra-low attachment 96-well plates in a volume of 100 μL (2 × 10^3^ glioblastoma and colorectal carcinoma cells and 2 × 10^3^ human fibroblasts per well). The plate was placed on a magnetic drive and incubated in a humidified atmosphere containing 5% CO_2_ at 37 °C until spheroids formed. After 2 days of incubation, the photos of spheroids were taken and the medium was replaced by the new medium. Photos were taken every 48 h, and the medium was replaced every 96 h. The effect of ursolic, oleanolic, betulinic, and corosolic acids in 3D glioblastoma and colorectal carcinoma cell cultures was analyzed by measuring the size change of spheroids using ImageJ software (National Institutes of Health).

### 2.13. Statistical Analysis

The statistical analysis of the research data was carried out using Microsoft Office Excel 2013 (Microsoft, Redmond, WA, USA) and SPSS 25.0 (SPSS Inc., Chicago, IL, USA) computer software. All the data gained during the HPLC analysis were provided as means of three successive test results and standard deviations. Univariate analysis of variance (ANOVA) was applied in order to estimate whether the variances among the compared data were statistically significant. The hypothesis about the equality of variances was tested by Levine’s test. If the variances of independent variables were estimated to be equal, Tukey’s multiple comparison test was applied. The differences were held as statistically significant at *p* < 0.05. To establish the linear dependence between the antiradical reductive activity and the anti-hyaluronidase and cytotoxic effects of apple extracts, the Pearson correlation coefficient was calculated. Pearson correlation coefficients: 0 < |r| ≤ 0.3 is a weak correlation; 0.3 < |r| ≤ 0.7 is a moderate correlation; 0.7 < |r| ≤ 1 is a strong correlation [33].

## 3. Results

### 3.1. Qualitative and Quantitative Analysis of Triterpene and Phenolic Compounds

**Figure 1:**
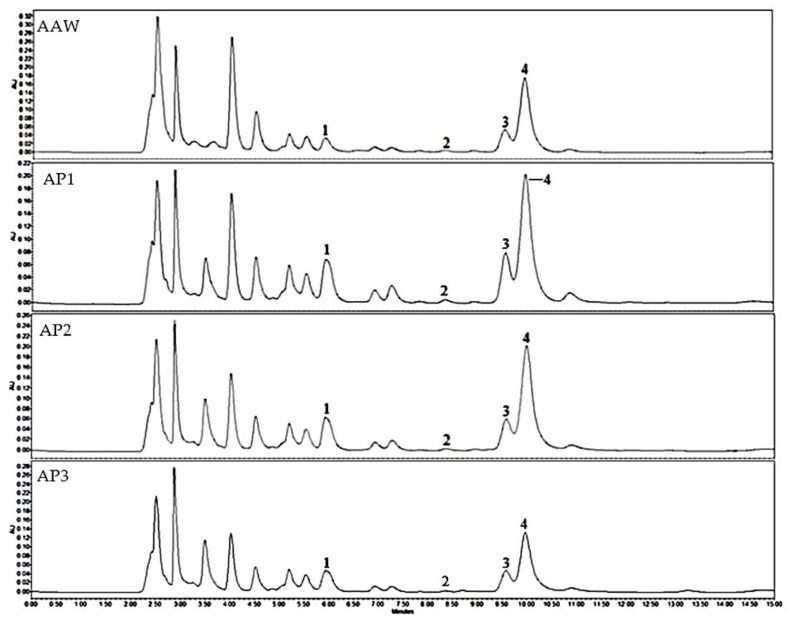
HPLC chromatogram of the apple acetone extracts. Analytes determined at λ = 205 nm: 1—corosolic acid; 2—betulinic acid; 3—oleanolic acid; 4—ursolic acid.

The performed study evaluated the variations in the qualitative and quantitative compositions of triterpenes of dry extracts from whole apple and apple peel. In apple samples, the following triterpenes were identified and qualitatively evaluated: betulinic acid, corosolic acid, oleanolic acid, and ursolic acid, respectively (Figure 1).

**Figure 2:**
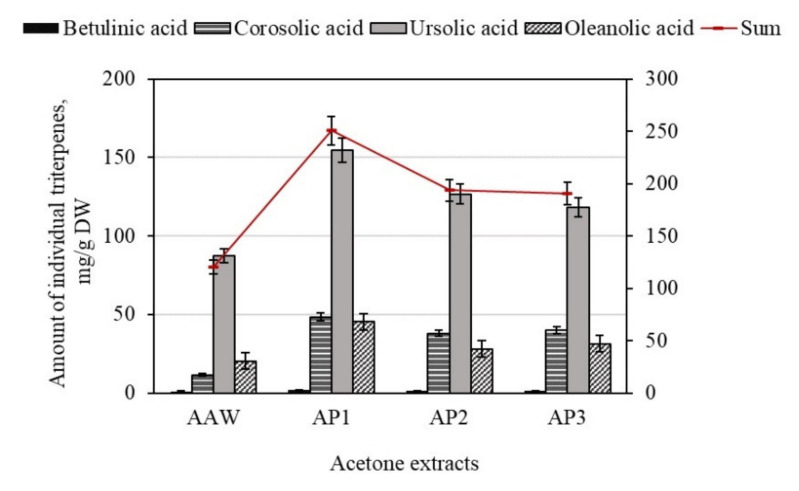
Variation in the quantitative amount of triterpene compounds in the dry acetone extracts of whole apple and apple peel.

The total amount of triterpene compounds in the dry apple acetone extracts varied from 120.26 ± 16.85 mg/g to 250.51 ± 21.03 mg/g (Figure 2). The highest total content of triterpene compounds (250.51 ± 21.03 mg/g) was found in the AP1 dry extract, with the lowest content (120.26 ± 16.85 mg/g) found in the AP3 dry extract (Figure 2). Hyson et al. have previously provided data showing that the contents of bioactive compounds varies between different parts of apples (i.e., it is peels and flesh) [34]. Apple fruit contains a high amount of lipophilic triterpene compounds mostly localized into the cuticular wax layer [35,36].

Quantitative analysis of individual triterpene compounds showed that ursolic acid was the dominant compound in the studied dry acetone extracts of the whole apple and apple peel. The highest content of ursolic acid (154.79 ± 17.23 mg/g) was found in the AP1 dry extract, which accounted for 61.8% of the total amount of all the determined triterpenes (Figure 2). The highest amount of oleanolic, corosolic, and betulinic acids was evaluated in the AP1 dry extracts, accounting for 18.1%, 19.3%, and 0.8% of the content of all detected triterpene compounds, respectively (Figure 2). Research by Sut et al. confirms the results established in our study, identifying ursolic acid as the most abundant compound [37]. It has been reported that ursolic acid may include 70% or more of the total content of triterpenes detected in apple fruits [38]. All the triterpene acids identified and quantified in the dry acetone extracts of the whole apple and apple peel can be ranked in the following order, ascending by content: ursolic acid < corosolic acid < oleanolic acid < betulinic acid.

**Figure 3:**
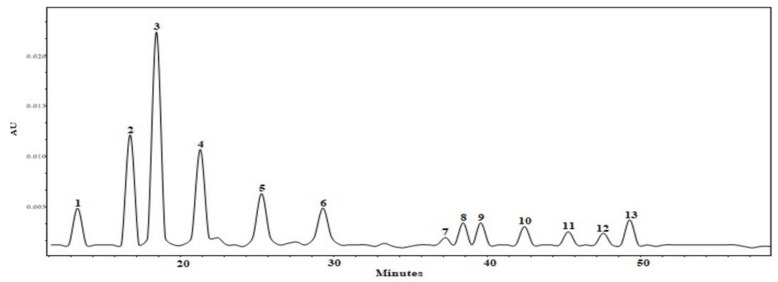
HPLC chromatogram of the dry ethanol extract of apple (AEW). Analytes determined at a λ = 280 nm wavelength: 1—procyanidin B1; 2—(+)-catechin; 3—chlorogenic acid; 4—procyanidin B2; 5—(−)-epicatechin; 6—procyanidin C1; at a λ = 360 nm wavelength: 7—rutin; 8—hyperoside; 9—isoquercitrin; 10—reynoutrin; 11—avicularin; 12—quercitrin; at a λ = 280 wavelength: 13—phloridzin.

Fruit extracts are multi-constituent matrices of bioactive compounds that vary in chemical structure and composition [39]. As such, it is important to determine not only the profiles of triterpenes, but also to identify and quantify the phenolic compounds that accumulate in apple fruits. The obtained results allowed for a correct estimation of the qualitative and quantitative compositions of individual phenols in the dry ethanol extracts of whole apple. Different groups of phenolic compounds were identified and quantified in the analyzed apple extracts: quercetin glycosides (rutin, hyperoside, isoquercitrin, reynoutrin, avicularin, and quercitrin), flavan-3-ols (procyanidin B1, procyanidin B2, procyanidin C1, (+)-catechin, and (−)-epicatechin), dihydrochalcones (phloridzin), and phenolic acids (chlorogenic acid) (Figure 3).

**Figure 4:**
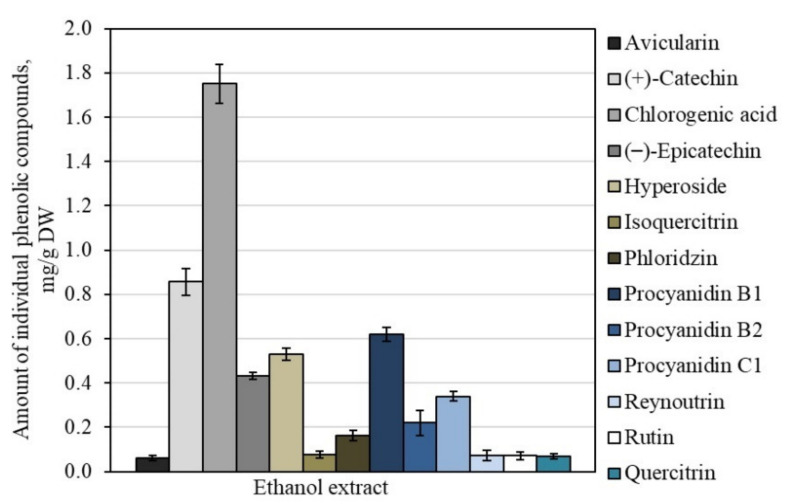
Variation in the quantitative amount of phenolic compounds in the dry extracts of whole apple.

The sum of the identified and quantifid individual phenolic compounds determined in the AEW dry extract of the whole apple was 5.26 ± 0.45 mg/g. Chlorogenic acid predominated among all the identified phenolic compounds at a volume of 1.75 ± 0.18 mg/g, accounting for 33.3% of the total amount of all the detected phenolic compounds (Figure 4). These results confirm those of the previous studies, that chlorogenic acid is one of the most predominant phenolic compounds in apples [40,41]. De Paepe et al. previously provided data showing that the content of chlorogenic acid in apple peel extracts varies from 0.07 mg/g to 1.38 mg/g [42].

Another group of flavan-3-ol compounds was identified and quantified in the apple extracts comprising monomeric compounds ((+)-catechin and (−)-epicatechin) and oligomeric compounds (procyanidin B1, procyanidin B2, and procyanidin C1). The total content of compounds in the flavan-3-ol group was 2.47 ± 0.23 mg/g, which accounted for 46.9% of the total content of the phenolic compounds (Figure 4). The total content of quercetin glycosides was 0.88 ± 0.11 mg/g, which included 16.7% of the total content of phenolic compounds detected in the dry extract of the whole apple (Figure 4). Hyson et al. have described that the content of quercetin glycosides detected in 67 different cultivars of apple samples varies from 0.08 mg/g to 1.66 mg/g [34]. Phloridzin, a compound of the dihydrochalcone group, was detected in the dry extract of the whole apple as well. Its quantitative content in the sample was 0.16 ± 0.08 mg/g, accounting for 3.1% of the total amount of phenolic compounds detected in the apple sample (Figure 4). De Paepe et al. have previously provided data showing that phloridzin content in apple peel samples varies from 0.03 to 0.73 mg/g [42].

The profiles of biologically active compounds in fruits vary according to different factors such as the cultivation environment, cultivar, plant part, and processing technique [43].

### 3.2. Antioxidant Activities of Whole Apple and Apple Peel Extracts

Evaluating antioxidants in foods (including vegetables and fruits) is important, as is evaluating total antioxidant activities in fruits by using different assays to obtain the exhaustive antioxidant potentials of their multi-component matrices. To achieve this, the total antioxidant activity of whole apple and apple peel extracts was estimated using four in vitro assays, namely, DPPH, ABTS, CUPRAC, and FRAP. The identified antioxidant mechanisms have important implications that can be used to better understand the protective effects of biologically active substances in apple extracts on cancer formation and inflammatory response.

Antiradical DPPH and ABTS assays explain the ability of antioxidants to scavenge free radicals. DPPH^•^ is a free radical soluble in organic solvents that restricts the estimation of antiradical activity in hydrophilic agents [44,45]. The ABTS^•+^ free radical is a cation soluble in water and organic solvents that the antiradical activity of hydrophilic and lipophilic agents to be assessed in weak alkaline conditions (pH = 7.4) [9].

**Figure 5:**
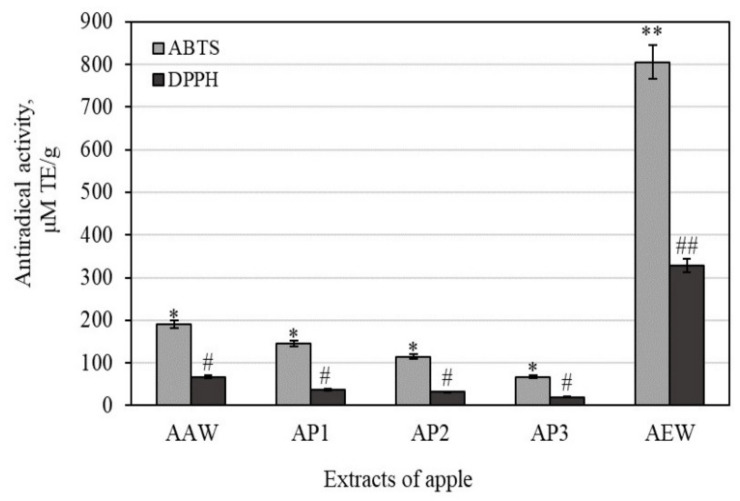
Antiradical activity of whole apple and apple peel extracts evaluated via ABTS and DPPH free radical scavenging assays. Different signs (* and #) indicate statistically significant differences at *p* > 0.05, according to Tukey’s multiple comparison test.

In our study, the strongest antiradical effects of scavenging DPPH^•^ free radicals (328.32 ± 24.58 μM TE/g) were determined in the AEW extract, while the weakest effects (19.37 ± 5.24 μM TE/g) were found in the AP3 extract (Figure 5). Faramarzi et al. found that the antiradical effects of scavenging DPPH^•^ free radicals varied from 10.11 to 129.09 μM TE/g among 67 apple extracts [46]. Han et al. evaluated the antiradical effects of scavenging DPPH^•^ free radicals in the crabapple fruit extracts and found them to vary from 120.36 to 383.19 μM TE/g [47].

The antiradical activity determined by the ABTS method confirms the results of the studies described above, that the strongest antiradical effects of scavenging ABTS^•+^ free radicals (805.52 ± 59.42 μM TE/g) were determined in the AEW extract, and the weakest effects (67.07 ± 11.42 μM TE/g) were determined in the AP3 extracts (Figure 5). Chen et al. carried out a parallel study to determine the antiradical activity of scavenging of ABTS^•+^ free radicals in apple extract [48]. Han et al. identified antiradical activities of scavenging ABTS^•+^ free radicals varying from 60.12 to 176.32 μM TE/g in crabapple fruit extracts [47].

The CUPRAC assay estimates reductive activity by measuring antioxidant potency and reducing Cu (II) to Cu (I) at a neutral pH value [30]. The FRAP assay aids in establishing the reductive activity by reducing Fe (III) to Fe (II) under acidic conditions (pH = 3.6) [29,49].

**Figure 6:**
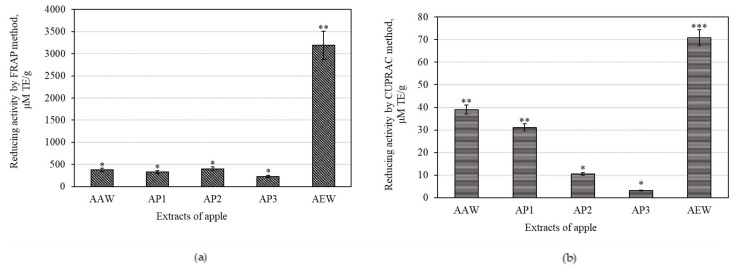
Reduction activity of the of whole apple and apple peel extracts: (**a**) reducing activity evaluated by the CUPRAC method; (**b**) reducing activity determined by the FRAP method. Different signs (*) indicate statistically significant differences at *p* > 0.05, according to Tukey’s multiple comparison test.

The strongest reduction activity evaluated by the CUPRAC assay (70.81 ± 12.88 μM TE/g) was detected in the AEW extract, while the weakest (3.24 ± 0.42 μM TE/g) was found in the AP3 extract (Figure 6a). Sethi et al. have previously identified CUPRAC reductive varying from 47.99 to 185.95 μM TE/g in apple peel extract [50].

The strongest reduction activity evaluated by the FRAP assay (3191.09 ± 110.12 μM TE/g) was detected in the AEW extract, with the weakest (229.83 ± 15.24 μM TE/g) discovered in the AP3 extract (Figure 6b). Previously data have shown 13 cultivars of apple FRAP values varying between 50.47–192.02 and 71.79–137.66 μM TE/g in the peel and cortex, respectively [50].

The whole apple ethanol extract (marked AEW) had four and five times stronger antiradical activity as determined by the ABTS and DPPH methods, and two and eight times stronger reducing activity as evaluated by the CUPRAC and FRAP methods, when compared to the apple peel acetone extracts marked AAW, AP1, AP2, and AP3, respectively. The antioxidant activities closely correlated with the contents of phenolic compounds [51]. Consequently, a decrease in antioxidant activity may be attributed to a decrease in phenolic compound content.

### 3.3. Anti-Inflammatory Activities of Apple Extracts and Individual Triterpenes

Inflammation is a biological process responding to infection, injury, or irritation [52]. Cancer initiation and development and most other chronic diseases including diabetes, obesity, cardiovascular, and neurologic illness are closely related to oxidative stress and chronic inflammation [14]. Hyaluronidase is an enzyme that depolymerizes the hyaluronic acid involved in inflammation, allergic reactions, and the migration of cancer cells [53,54]. Omar et al. have shown that the inhibition of hyaluronidase and subsequent reduction in the breakdown of hyaluronic acid result in a decrease in angiogenesis and inflammation [55]. However, the whole apple and apple peel extracts and the most dominant triterpenes that are useful as anti-inflammatory agents (creating a hyaluronidase inhibitory effect) have not been published.

**Figure 7:**
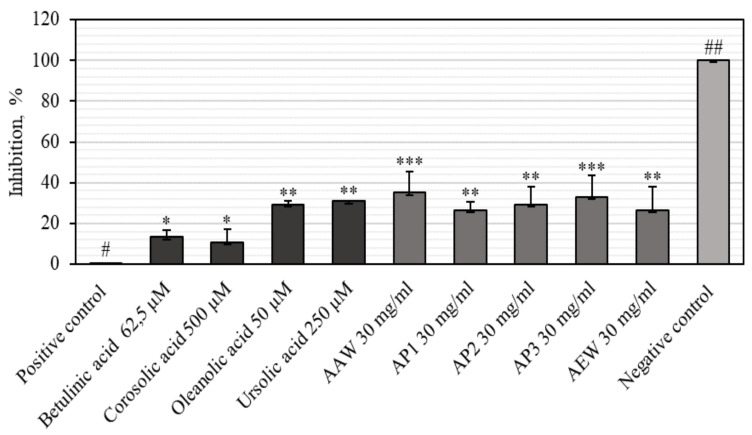
Hyaluronidase inhibition effect of apple extracts and individual triterpenes. Different signs (* and #) indicate statistically significant differences at *p* > 0.05, according to Tukey’s multiple comparison test.

Our results show that the apple extracts inhibited hyaluronidase from 26.38 ± 4.4% to 35.05 ± 3.8% (Figure 7). The strongest inhibition activity of hyaluronidase (35.05 ± 3.8%) was evaluated in the AAW extract (Figure 7). Meanwhile, the weakest inhibition effect of hyaluronidase (26.38 ± 4.4%) was determined in the AEW extract (Figure 7). Lee et al. found that the Summer King and Fuji apple peel ethanol extracts had a hyaluronidase inhibition effect that varied from 4.56% to 20.40%, and from 1.29% to 12.72%, respectively [56]. Piwowarskia et al. determined that *Quercus robur* L. bark (20.2 ± 2.2%), *Rubus idaeus* L. leaf (21.2 ± 2.0%), *Geum urbanum* L. root (25.5 ± 5.1%), and *Lythrum salicaria* L. aerial part (64.9 ± 6.3%) extracts inhibit hyaluronidase [57]. A previous study has also revealed the presence of hyaluronidase inhibitors in *Punica granatum* L. fruits and *Camellia sinensis* extracts [58].

High hyaluronidase inhibitory effects of 30.93 ± 0.61% and 29.15 ± 0.89% were observed in ursolic and oleanolic acids, respectively (Figure 7). The weakest hyaluronidase inhibition effect (10.90 ± 2.1%) was found in corosolic acid (Figure 7). Abdullah et al. previously performed a structural activity analysis evaluating the anti-hyaluronidase effect of ursolic and oleanolic acids and their derivatives. The results of the study revealed that ursolic acid was more active than oleanolic acid [54]. Abdullah et al. determined that the hydroxyl groups at C-3 and C-28 were significant for both ursane and oleanane skeletons of pentacyclic triterpenes to inhibit hyaluronidase [54].

We evaluated the correlation coefficient between the antioxidant and hyaluronidase inhibition activities of apple extracts. A moderate positive correlation (r_DPPH_ = 0.397, r_ABTS_ = 0.422, r_FRAP_ = 0.420, and r_CUPRAC_ = 0.449) between the antioxidant and hyaluronidase inhibition activities of apple extracts was found. This correlation suggests that anti-inflammatory activity could be involved in the antioxidant properties of the studied compounds.

### 3.4. Cytotoxic Activity of Apple Extracts and Individual Triterpenes

#### 3.4.1. Cell Viability Determined by MTT Assays

A wide array of in vitro and in vivo studies have evaluated the potential of various antioxidant and anti-inflammatory agents to prevent carcinogenesis. In fact, many biologically active compounds of fruits and vegetables can protect cells or tissues from harmful oxidative and inflammation-mediated tissue damage, and consequently can prevent the development of cancer. In tumor development, hyaluronic acid, a main constituent of the extracellular matrix, plays an relevant role in a variety of cellular processes such as proliferation, adhesion, migration, invasion, metastasis, and drug resistance [18]. A large number of studies have shown that the volume of hyaluronic acid in tumors is usually higher in cancerous than in normal tissues. Colon cancer and glioblastoma, among other kinds of cancer, are enriched with hyaluronic acid [17,18,24]. In that type of cancer, hyaluronic acid may induce tumor development by stimulating anchorage-independent growth and the proliferation of tumor cells. In our studies, the cytotoxic activity of apple extracts and predominant individual triterpenes was detected.

**Figure 8:**
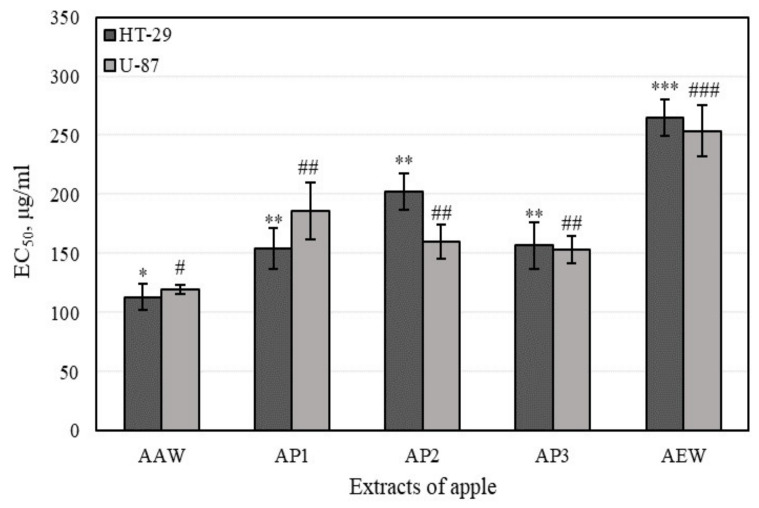
The effect of apple extracts on cell viability evaluated by MTT assay. Different signs (* and #) indicate statistically significant differences at *p* > 0.05, according to Tukey’s multiple comparison test.

The whole apple and apple peel extracts reduced the viability of colon adenocarcinoma (HT-29) and human glioblastoma (U-87) lines (Figure 8). The whole apple extract, (marked AEW) possessed the lowest (*p* < 0.05) cytotoxic activity against all cell lines (EC_50_ varied from 265.0 ± 15.1 to 253.7 ± 21.8 µg/mL against different cell lines). The whole apple extract (marked AAW) possessed the strongest cytotoxic activity (EC_50_ varied from 113.3 ± 11.11 to 119.7 ± 4.0 µg/mL, respectively, against different cell lines). A majority of the tested apple extracts (marked AP2, AP3, and AEW) were more active against human glioblastoma cells and demonstrated lower activity against colon cancer cell line.

Based on average values, cytotoxic activity against different cell lines was strongest in the following order: AAW < AP3 = AP1 = AP2 < AEW. Whole apple extract (marked AEW) was strongest, while phenolic compounds had the lowest cytotoxic activity. Shukla et al. have suggested that the various effects of phenolic compounds in cancer cell lines depend on cell prototypes (e.g., breast cancer cells with different epithelial growth factor receptors (HER2/neu) have different responses to phenolic compound treatments) [59]. Stump et al. evaluated the effect of phenolic compounds on cancer cells, determining that their activities are dependent on concentration (e.g., phenols diminished U-87 cell viability at concentrations lower than 100 µM). Our results and the literature data could depend on the differences between the cancer cell lines and the experimental conditions (e.g., medium pH) [60]. 

Although higher values of antioxidant and anti-hyaluronidase activities describe higher effects, and higher values of cell viability assay (expressed as EC_50_) explain lower activity, the moderate correlation (r_H-29_ = 0.331, and r_U-97_ = 0.669) between the anti-hyaluronidase and cytotoxic activities of apple extracts was evaluated (Table 2). The strong correlation between antioxidant and cytotoxic activities of apple extracts was determined and the results are shown in Table 2.

**Figure 9:**
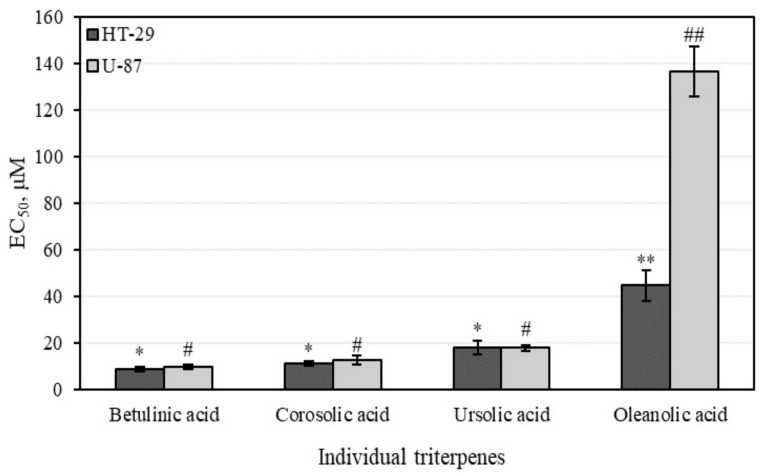
The effect of individual triterpenes on cell viability evaluated by MTT assay. Different signs (* and #) indicate statistically significant differences at *p* > 0.05, according to Tukey’s multiple comparison test.

The study data provided in Figure 9 demonstrate cytotoxic activity of major triterpenes detected in apple extracts against colon adenocarcinoma (HT-29) and human glioblastoma (U-87) lines. Oleanolic acid reduced the viability of cancer cells lines the least, by 50% (EC_50_ varied from 44.7 ± 6.8 and 136.7 ± 10.6 µM against different cell lines). Betulinic acid showed the strongest cytotoxic activities (EC_50_ varied from 8.9 ± 0.7 to 9.9 ± 0.2 µM against different cell lines). The individual triterpenes betulinic, corosolic, and oleanolic acids were more active against human colon cancer cells and showed lower activity against glioblastoma cell line. A previous study showed that betulinic and oleanolic acids reduce the viability of HT-29 cancer cells in a dose-dependent manner [61]. Juan et al. determined that incubation of HT-29 cells for 72 h with 50 and 200 mmol/L oleanolic acid induced 16% and 62% reductions in cell numbers respectively, with an overall EC_50_ of 160.6 ± 10.6 µM [61]. Bache et al. found that betulinic acid reduced the viability of malignant glioma cells (EC_50_ varied from 16.8 to 20.0 µM) [62].

Ursolic acid moderately reduced cell viability in human colon adenocarcinoma (HT-29) and human glioblastoma (U-87) cell lines. The EC_50_ values determined in HT-29 and U-87 cells were 18.1 ± 3.2 µM and 17.9 ± 0.2 µM, respectively (Figure 9). Wang et al. found that ursolic acid exerts an inhibitory effect on U-251 glioma cells in a time-dependent manner [63]. Shen et al. determined that ursolic acid reduced U-87MG cell viability to an EC_50_ value of 45.7 µM [64]. Tan and Andersson found that ursolic acid activated intestinal alkaline sphingomyelinase in HT-29 human colon carcinoma cells in vitro and in vivo [65,66]. Shan et al. determined that ursolic acid may inhibit HT-29 cell viability and induce apoptosis through the EGFR/MAPK signal pathway [67]. HT-29 cells treated with 10, 20, and 40 μM ursolic acid showed significant dose-dependent inhibition, with cell proliferation rates decreased by 38.5%, 65.9%, and 78.4%, respectively, compared with EGF-stimulated cells. Shan et al. demonstrated that ursolic acid inhibits the proliferation of HT-29 cells by suppressing EGFR phosphorylation [67]. In that study, ursolic acid dose-dependently decreased the phosphorylation of p38 MAPK and JNK and reached an almost-complete inhibition at 40 μM. Ursolic acid inhibited the phosphorylation of ERK1/2, p38 MAPK, and JNK in both dose- and time-dependent ways, and reduced HT-29 cell viability [67]. Shan et al. found that ursolic acid inhibits HT-29 cell proliferation and induces apoptosis through the activation of caspase-3, -8, and -9 [67].

We found that individual triterpene compounds had stronger cell viability-reducing activity compared to multicomponent apple extracts. Based on their average cytotoxic activity values, individual triterpenes showed cytotoxic activities against different cell lines in the following order: betulinic acid < corosolic acid < ursolic acid < oleanolic acid. The stronger cell viability-reducing effect of individual triterpenes may have been offset by faster and better absorption into cancer cells. More detailed research is needed to assess the cytotoxic activity of individual triterpenes.

#### 3.4.2. Effect of Individual Triterpenes on HT-29 Colony-Forming

**Figure 10:**
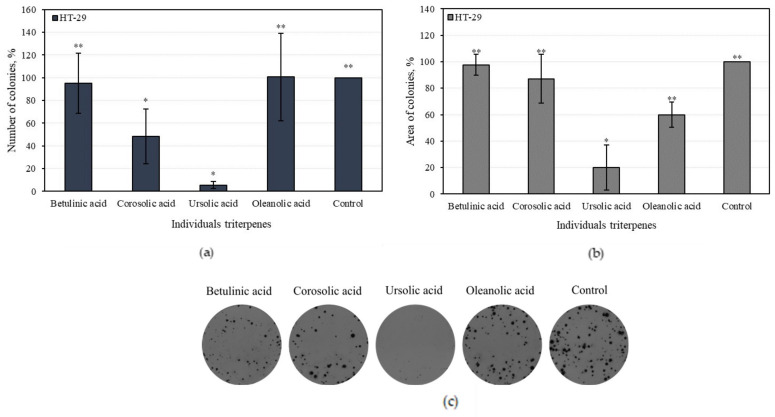
The effect of individual triterpenes on HT-29 colony-forming: (**a**) numbers of colonies; (**b**) area of colonies; (**c**) colonies incubated with different triterpenes. Different signs (*) indicate statistically significant differences at *p* > 0.05, according to Tukey’s multiple comparison test.

Human colon adenocarcinoma (H-29) cell colony growth was evaluated by comparing the number and total area of colonies incubated in the medium with individual triterpene compounds. Colonies in the control group were equal to 100%. In the medium with ursolic acid, the number of HT-29 colonies was the lowest and accounted for 5.5 ± 4.8% (*p* < 0.05) (Figure 10a). Oleanolic and betulinic acids did not reduce the number of colonies with statistical significance (Figure 10a).

In the present study, we found individual triterpenes reduced the total areas of colonies. The area of one colony of the control group was equal to 100%. The areas of the colonies incubated with ursolic and oleanolic acid were 20% and 60%, respectively (Figure 10b). Corosolic and betulinic acids did not statistically significantly incubate the areas of colonies compared to the control group (Figure 10b).

Ursolic acid was the most effective triterpene in reducing the number of colonies, as it incubated the colony areas with more than five times greater efficiency than the control area. Oleanolic acid weakly incubated the total amount of colonies; however, it reduced the areas of the colonies.

#### 3.4.3. Triterpenes on U-87 Cell 3D Spheroid Growth

**Figure 11:**
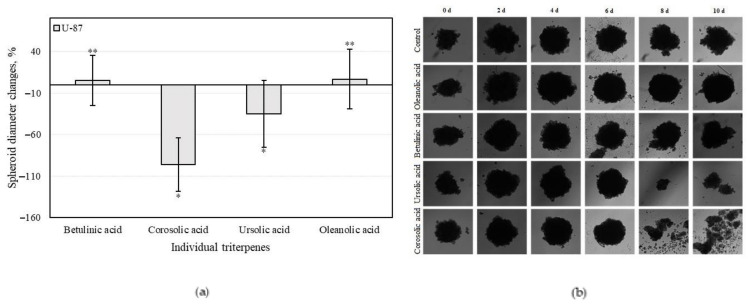
The effect of individual triterpenes on U-87 cell spheroid growth: (**a**) spheroid diameter changes; (**b**) spheroid disintegration. Different signs (*) indicate statistically significant differences at *p* > 0.05, according to Tukey’s multiple comparison test.

An analysis of 3D cell cultures showed that human glioblastoma (U-87) cell spheroids can disintegrate in the presence of triterpene compounds. Negative spheroid growth change (Figure 11a) and the breakdown of these spheroids (Figure 11b) were observed when U-87 cell spheroids were incubated with corosolic and ursolic acids (Figure 11a).

Of all the triterpenes tested, corosolic acid most efficiently reduced the growth of spheroids. Nine out of the U-87 spheroids disintegrated after 10 days incubation with corosolic acid, indicating an estimated −100% change in spheroid size. Ursolic acid reduced spheroid growth by 40% compared to the control. After 10 days incubation with ursolic acid, 6 out of 10 spheroids had disintegrated. Spheroids affected by betulinic and oleanolic acids remained in contact and even slightly enlarged after 10 days incubation compared to control group.

In our study, we found that two triterpene acids, namely, corosolic and ursolic, reduced the growth and initiated breakdown of U-87 spheroids; meanwhile, oleanolic and betulinic acid slightly promoted their growth.

## 4. Discussion

When the natural protection system of an organism including enzymatic, non-enzymatic, or dietary origins is related to the exaggerated generation of reactive oxygen and nitrogen species, macromolecules can suffer oxidative damage, inducing tissue damage [68]. Intake of foods (especially fruits) with a lot of antioxidant agents is an effective strategy to contend with such tissue damage and objectionable transformations, and can prevent the development of chronic diseases.

The total antioxidant activities of the whole apple and apple peel extracts were estimated using four in vitro assays, namely, DPPH and ABTS free radical scavenging and copper (CUPRAC) and ferric (FRAP) reducing activities. The identified antioxidant mechanisms have important implications for the protective effects of biologically active substances of apple extracts on inflammatory response and cancer formation. Antioxidant activity is closely correlated with phenolic compound content [51]. Consequently, a decrease in antioxidant activity of apple acetone extract may be attributed to a decrease in phenolic compound content.

Human cells are constantly affected by oxidative stresses, and a deficiency in antioxidants can lead to the hoarding of reactive oxygen species, causing cell damage, fibrosis, and cell proliferation, all of which can contribute to chronic inflammation [69]. This chronic inflammation, which remains even after the removal of the pathogen, has been connected to several diseases such as cancer, neoplasms, inflammatory bowel disease, ulcerative colitis, atherosclerosis, rheumatoid arthritis, asthma, and Alzheimer’s disease [70]. The injurious responses resulting from chronic inflammation can be controlled by changing the molecular mediators of an inflammatory response. One of the most relevant enzymes in inflammatory response is hyaluronidase, which depolymerizes hyaluronic acid and controls of the molecular size and contents of hyaluronic acid chains, thereby modulating the activity of many pathological effects that strongly depend on their length [22,23]. Whatcott et al. has described how hyaluronic acid is involved in cell proliferation, tissue hydration, cell mobility, inflammation, angiogenesis, and malignancy [71]. The study of hyaluronidase inhibitors as potent regulating compounds is important, as they are involved in maintaining the homeostasis of hyaluronic acid.

In our study, we found that apple extracts inhibited hyaluronidase from 26.38 ± 4.4% to 35.05 ± 3.8%. A previous study showed that hyaluronidase inhibitors had different chemical forms and plant-derived bioactive components, namely, glycosaminoglycans, polysaccharides, fatty acids, alkaloids, phenolic acids, flavonoids, and triterpenes [25]. Liyanaarachchi et al. discovered strong hyaluronidase inhibition activities in *Curucumaa aromatica* (95.02%), *Azadirachta indica* (65.64%), *Artocarpus altilis* (68.59%), and *Artocarpus heterophyllus* (52.62%) extracts [72]. Our study showed that individual triterpene compounds inhibited hyaluronidase from 10.90 ± 2.1% to 30.93 ± 0.61%. Pentacyclic triterpenes such as ursolic, oleanolic, betulinic, and corosolic acid are contained in apple fruits and throughout the plant kingdom, and are characterized by a wide range of biological effects [73,74]. Triterpene acids decrease inflammation in various mechanisms (e.g., by inhibiting enzymes in relation to inflammatory response) [74]. Triterpenes not only have a hyaluronidase inhibiting effect, they also may inhibit lipoxygenase, phospholipase A2, cyclooxygenase (COX), nitric oxide synthase, and elastase [75]. Moreover, triterpene acids can reduce the formation of pro-inflammatory mediators such as prostaglandins and cytokines [76]. The potency of ursolic acid to attenuate expression of COX-2 and the secretion of pro-inflammatory cytokines such as tumor necrosis factor α (TNF-α), interferon γ (IFN-γ), and interleukins (e.g., IL-6) is also associated with its cytotoxic activity, since chronic inflammation is recognized as a cancerogenesis-promoting condition [77]. Based on the results they obtained, Girish et al. claimed that in the structure of pentacyclic triterpenes, the double bond between C-2 and C-3 positions is critical for hyaluronidase inhibition. However, the keto group at position C-4 and different substituents would seem relevant to the inhibitory effect of the compounds. The introduction of hydroxyl groups in positions C-5, C-7, and C-4 increase the inhibitory potency [25].

Tumor development is closely related to oxidative stress and chronic inflammation [14]. Previous studies have shown that hyaluronic acid interacts with CD44, relieving colon, breast, and brain cancer cell migrations. The content of hyaluronic acid itself correlates with overall cancer aggressiveness and increased cell migration and proliferation in breast and ovarian cancer. Moreover, high hyaluronic acid content has been correlated with poor prognosis in gastric, colorectal, breast, ovarian, and bladder cancers [14].

The cytotoxic activity of apple extracts and the predominant individual triterpene compounds was herein evaluated. Whole apple and apple peel extracts reduced the viability of colon adenocarcinoma (HT-29) and human glioblastoma (U-87) lines. The majority of apple extracts tested, namely, AP2, AP3, and AEW, were more active against human glioblastoma cells and showed lower activity against colon cancer cell lines. The weakest cytotoxic activity (*p* < 0.05) was evaluated in the extract of whole apple (AEW) comprising predominately phenolic compounds. Meanwhile, the strongest cytotoxic activity was evaluated in the extract of whole apple (AAW) comprising predominately triterpenes.

The study of human colon adenocarcinoma (HT-29) and human glioblastoma (U-87) cell viability were determined for individual triterpenes. We found that individual triterpene compounds had stronger cell viability-reducing activities compared to multicomponent apple extracts. The stronger cell viability-reducing effects of individual triterpenes may have been offset by faster and better absorption into cancer cells. Physicochemical properties of the compounds should be discussed, such as molecular weight, H-bonding with solvents, intramolecular H-bonding, intermolecular H-bonding, crystallinity, rate of dissolution, polymorphic forms, salt form, and ionic charge status [78,79]. Greater knowledge about physicochemical properties of bioactive compounds in foods, including fruits, might help develop an understanding of their interactions with complex matrices [80]. THE human colon adenocarcinoma (H-29) cell colony growth was detected by comparing the number and total area of colonies incubated in the medium with individual triterpene compounds. Ursolic acid was the most effective at reducing the number of colonies and incubated the colony areas with five times greater efficiency compared to the control area. Oleanolic acid weakly incubated numbers of colonies, however it reduced the areas of the colonies. An analysis of 3D bioprinting showed that human glioblastoma (U-87) cell spheroids affected with triterpene compounds could disintegrate. Corosolic acid was the strongest of all the tested triterpenes at reducing spheroid growth. Of 10 U-87 cells affected by corosolic acid, nine of them disintegrated after 10 days, suggesting an estimated −100% change in spheroid size. Betulinic and oleanolic acid-affected spheroids remained healthy and elevated after 10 days compared to the control groups.

Apple extracts with triterpene complexes, namely, AAW, AP1, AP2, and AP3, inhibited hyaluronidase and reduced human colon adenocarcinoma (H-29) and human glioblastoma (U-87) cell viabilities the best compared with apple extract marked AEW, in which phenolic compounds predominated. Previous studies have revealed anticancer activities of some hyaluronidase inhibitors. For example, glycyrrhizin, a hyaluronidase inhibitor also known as anti-inflammatory agent, reduced the growth, migration, and metastasis of glioblastoma, lung cancer, and leukemia [22]. A previous study showed that inactivation of the PI3K/Akt/mTOR pathway could be a possible mechanism underlying the anticancer activities of hyaluronidase inhibitors [22].

The antioxidative and anti-inflammatory activities of apple extracts and individual triterpenes are potentially linked to their chemopreventive efficacy. Triterpene acids, as hyaluronidase inhibitors, can be used as a cancer biomarker and potential therapeutic target. Understanding the mechanisms of hyaluronidases related to tumor progression could be a potential tool for the prognosis of colon cancer and glioblastoma. However, how such regulation and mechanisms are accomplished remains to be clarified.

## 5. Conclusions

The antioxidant, anti-inflammatory, and cytotoxic activities of apple extracts were evaluated. We found that the whole apple (AEW) ethanol extract had four and five times stronger antiradical activity as determined by the ABTS and DPPH methods, and two and eight times stronger reducing activity as evaluated by the CUPRAC and FRAP methods, compared to the apple acetone extracts AAW, AP1, AP2, and AP3, respectively. The anti-inflammatory study showed that the strongest inhibition effect of hyaluronidase (35.05 ± 3.8%) in the whole apple (AAW) acetone extract. The cytotoxic activity of apple extracts and predominant individual triterpene compounds was identified. The majority of the tested apple extracts marked AP2, AP3, and AEW were more active against human glioblastoma (U-87) cells, and showed lower activity against colon cancer (HT-29) cell line. Betulinic acid showed the strongest cytotoxic activities (EC_50_ varied from 8.9 ± 0.7 to 9.9 ± 0.2 µM against different cell lines). The individual triterpenes, namely, betulinic, corosolic, and oleanolic acids, were more active against human colon cancer cells and showed lower activity against glioblastoma cell lines. Ursolic acid was the most effective at reducing the number of colonies, and even incubated the colony areas with five times greater efficiency compared to the control area. An analysis of 3D bioprinting showed that human glioblastoma cell spheroids can disintegrate in the presence of triterpene compounds. Corosolic acid had the strongest capacity to reduce spheroid growth and initiate spheroid disintegration compared with the other triterpenes.

The antioxidative, anti-inflammatory, and cytotoxic activities of apple extracts and individual triterpenes have important implications that could allow us to better understand the biological effects of apple extracts. Understanding the mechanisms of apple extracts and individual triterpenes as hyaluronidase inhibitors and antioxidants involved in cancer progression may be a useful tool for future study in vivo, cancer prognosis, or development of new, innovative food supplements that could be used for chronic diseases prevention.

## Figures and Tables

**Table 1:** The study used apple samples and their abbreviations.

No.	Cultivars	Dry Extracts	Abbreviation
1.	‘Kostele’	Whole apple	AAW
2.	‘Ligol’	Apple peel	AP1
3.	‘Rubin’	Apple peel	AP2
4.	‘Auksis’	Apple peel	AP3
5.	‘Paprastasis antaninis’	Whole apple	AEW

**Table 2:** Correlation among antioxidant, anti-hyaluronidase, and anti-proliferative activity in apple extracts.

		DPPH	ABTS	CUPRAC	FRAP	HT-29	U-87
Anti-HYAL	Pearson Correlation	0.397	0.422	0.420	0.449	0.331	0.669
	Sig. (2-tailed)	0.508	0.479	0.482	0.448	0.587	0.217
HT-29	Pearson Correlation	0.728	0.729	0.427	0.796	1	0.904 *
	Sig. (2-tailed)	0.163	0.162	0.473	0.107		0.035
U-87	Pearson Correlation	0.835	0.846	0.658	0.881 *	0.904 *	1
	Sig. (2-tailed)	0.079	0.071	0.227	0.049	0.035	

*—Correlation is significant at the 0.05 level (2-tailed).

## Data Availability

All datasets generated for this study are included in the article.

## References

[1] Food and Agriculture Organization FAOSTAT. http://www.fao.org/faostat/en/#data/QL.

[2] Wu J., Gao H., Zhao L., Liao X., Chen F., Wang Z., Hu X. (2007). Chemical compositional characterization of some apple cultivars. Food Chem..

[3] Morresi C., Cianfruglia L., Armeni T., Mancini F., Tenore G.C., D’Urso E., Micheletti A., Ferretti G., Bacchetti T. (2018). Polyphenolic compounds and nutraceutical properties of old and new apple cultivars. J. Food Biochem..

[4] Oszmianski J., Lachowicz S., Gławdel E., Cebulak T., Ochmian I. (2017). Determination of phytochemical composition and antioxidant capacity of 22 old apple cultivars grown in Poland. Eur. Food Res. Technol..

[5] Ferretti G., Turco I., Bacchetti T. (2014). Apple as a source of dietary phytonutrients: Bioavailability and evidence of protective effects against human cardiovascular disease. Food Nutr. Sci..

[6] Nour V., Trandafir I., Ionica M.E. (2010). Compositional characteristics of fruits of several apple *(Malus domestica* Borkh.) cultivars. Not. Bot. Hort. Agrobot..

[7] Berni R., Cantini C., Guarnieri M., Nepi M., Hausman J.-F., Guerriero G., Romi M., Cai G. (2019). Nutraceutical characteristics of ancient *Malus × domestica* Borkh. fruits recovered across Siena in Tuscany. Medicines.

[8] Boyer J., Liu R.H. (2004). Apple phytochemicals and their health benefits. Nutr. J..

[9] Pandey K.B., Rizvi S.I. (2009). Plant polyphenols as dietary antioxidants in human health and disease. Oxid. Med. Cell. Longev..

[10] Allouche Y., Beltrán G., Gaforio J.J., Uceda M., Mesa M.D. (2010). Antioxidant and antiatherogenic activities of pentacyclic triterpenic diols and acids. Food Chem. Toxicol..

[11] Jeong J.W., Shim J.J., Choi I.D., Kim S.H., Ra J., Ku H.K., Lee D.E., Kim T.Y., Jeung W., Lee J.H. (2015). Apple pomace extract improves endurance in exercise performance by increasing strength and weight of skeletal muscle. J. Med. Food.

[12] Waldbauer K., Seiringer G., Nguyen D.L., Winkler J., Blaschke M., McKinnon R., Urban E., Ladurner A., Dirsch V.M., Zehl M. (2016). Triterpenoic acids from apple pomace enhance the activity of the endothelial nitric oxide synthase (eNOS). J. Agric. Food Chem..

[13] Gerhauser C. (2008). Cancer chemopreventive potential of apples, apple juice, and apple components. Planta Med..

[14] Zhang C., Wang C., Li W., Wu R., Guo Y., Cheng D., Yang Y., Androulakis I.P., Kong A.N. (2017). Pharmacokinetics and pharmacodynamics of the triterpenoid ursolic acid in regulating the anti-oxidant, anti-inflammatory and epigenetic gene responses in rat leukocytes. Mol. Pharm..

[15] Chen C., Kong A.N. (2004). Dietary chemopreventive compounds and ARE/EpRE signaling. Free Radic. Biol. Med..

[16] Baldelli G., De Santi M., Fraternale D., Brandi G., Fanelli M., Schiavano G.F. (2019). Chemopreventive potential of apple pulp callus against colorectal cancer cell proliferation and tumorigenesis. J. Med. Food.

[17] Hartheimer J.S., Park S., Rao S.S., Kim Y. (2019). Targeting hyaluronan interactions for glioblastoma stem cell therapy. Cancer Microenviron..

[18] Chen J.W.E., Pedron S., Shyu P., Hu Y., Sarkaria J.N., Harley B.A.C. (2018). Influence of hyaluronic acid transitions in tumor microenvironment on glioblastoma malignancy and invasive behavior. Front. Mater. Sci..

[19] Kimlin C., Casagrande G., Virador V.M. (2013). In vitro threedimensional (3D) models in cancer research: An update. Mol. Carcinog..

[20] Herrera-Perez H.M., Voytik-Harbin S.L., Rickus J.L. (2015). Extracellular matrix properties regulate the migratory response of glioblastoma stem cells in three-dimensional culture. Tissue Eng. Part A.

[21] Lv D., Yu S.C., Ping Y.F., Wu H., Zhao X., Zhang H., Cui Y., Chen B., Zhang X., Dai J. (2016). A three-dimensional collagen scaffold cell culture system for screening anti-glioma therapeutics. Oncotarget.

[22] Kohi S., Sato N., Koga A., Hirata K., Harunari E., Igarashi Y. (2016). Hyaluromycin, a novel hyaluronidase inhibitor, attenuates pancreatic cancer cell migration and proliferation. J. Oncol..

[23] Stern R., Asari A.A., Sugahara K.N. (2006). Hyaluronan fragments: An informationrich system. Eur. J. Cell Biol..

[24] Bouga H., Tsouros I., Bounias D., Kyriakopoulou D., Stavropoulos M.S., Papageorgakopoulou N., Theocharis D.A., Vynios D.H. (2010). Involvement of hyaluronidases in colorectal cancer. BMC Cancer.

[25] Girish K.S., Kemparaju K., Nagaraju S., Vishwanath B.S. (2009). Hyaluronidase inhibitors: A biological and therapeutic perspective. Curr. Med. Chem..

[26] Butkevičiūtė A., Liaudanskas M., Kviklys D., Zymonė Z., Raudonis R., Viškelis J., Uselis N., Janulis V. (2018). Detection and analysis of triterpenic compounds in apple extracts. Int. J. Food Prop..

[27] Liaudanskas M., Viskelis P., Kviklys D., Raudonis R., Janulis V. (2015). A comparative study of phenolic content in apple fruits. Int. J. Food Prop..

[28] Brand-Williams W., Cuvelier M.E., Berset C. (1995). Use of a free radical method to evaluate antioxidant activity. Food Sci. Technol..

[29] Benzie I.F.F., Strain J.J. (1998). Ferric reducing/antioxidant power assay: Direct measure of total antioxidant activity of biological fluids and modified version for simultaneous measurement of total antioxidant power and ascorbic acid concentration. Methods Enzymol..

[30] Özyürek M., GüçLü K., Tütem E., Başkan K.S., Erçağ E., Çelik S.E., Baki S., Yıldız L., Karaman Ş., Apak R. (2011). A comprehensive review of CUPRAC methodology. Anal. Methods.

[31] El-Guendouz S., Aazza S., Lyoussi B., Antunes M.D., Faleiro M.L., Miguel M.G. (2016). Anti-acetylcholinesterase, antidiabetic, anti-inflammatory, antityrosinase and antixanthine oxidase activities of Moroccan propolis. Int. J. Food Sci. Technol..

[32] Grigalius I., Petrikaite V. (2017). Relationship between antioxidant and anticancer Activity of trihydroxyflavones. Molecules.

[33] Čekanavičius V., Murauskas G. (2014). Applied Regression Analysis in Social Research.

[34] Hyson D.A. (2011). A comprehensive review of apples and apple components and their relationship to human health. Adv. Nutr..

[35] Siani A.C., Nakamura M.J., Santos D.S., Mazzei J.L., Nascimento A.C., Valente L.M.M. (2014). Efficiency and selectivity of triterpenic acid extraction from decoctions and tinctures prepared from apple peels. Pharmacogn. Mag..

[36] Farneti B., Masuero D., Costa F., Magnago P., Malnoy M., Costa G., Vrhovsek U., Mattivi F. (2015). Is there room for improving the nutraceutical composition of apple?. J. Agric. Food Chem..

[37] Sut S., Zengin G., Maggi F., Malagoli M., Dall’Acqua S. (2019). Triterpene acid and phenolics from ancient apples of Friuli Venezia Giulia as nutraceutical ingredients: LC-MS study and in vitro activities. Molecules.

[38] Poirier B.C., Buchanan D.A., Mattheis J., Rudell D. (2018). Differential partitioning of triterpenes and triterpene esters in apple peel. J. Agric. Food Chem..

[39] Contessa C., Botta R. (2016). Comparison of physicochemical traits of red-fleshed, commercial and ancient apple cultivars. Hort. Sci..

[40] Tsao R., Yang R., Young A.J.C., Zhu H. (2003). Polyphenolic profiles in eight apple cultivars using high-performance liquid chromatography (HPLC). J. Agric. Food Chem..

[41] Duda-Chodak A., Tarko T., Satora P., Sroka P., Tuszy’nski T. (2010). The profile of polyphenols and antioxidant properties of selected apple cultivars grown in Poland. J. Fruit Ornam. Plant Res..

[42] De Paepe D., Valkenborg D., Noten B., Servaes K., Diels L., De Loose M. (2015). Variability of the phenolic profiles in the fruits from old, recent and new apple cultivars cultivated in Belgium. Metabolomics.

[43] Simmonds M.S.J., Howes M.J.R. (2016). Profile of compounds in different cultivars of apple (*Malus × domestica*). Nutritional Composition of Fruit Cultivars.

[44] Raudonė L., Raudonis R., Liaudanskas M., Janulis V., Viskelis P. (2017). Phenolic antioxidant profiles in the whole fruit, flesh and peel of apple cultivars grown in Lithuania. Sci. Hortic..

[45] Liaudanskas M., Zymone K., Viskelis J., Kviklys D., Viskelis P., Janulis V. (2018). Seasonal variation of the qualitative and quantitative composition of phenolic compounds in Malus domestica leaves. Chem. Nat. Comp..

[46] Faramarzi S., Pacifico S., Yadollahi A., Lettieri A., Nocera P., Piccolella S. (2015). Red-fleshed apples: Old autochthonous fruits as a novel source of anthocyanin antioxidants. Plant Foods Hum. Nutr..

[47] Han M., Li G., Liu X., Li A., Mao P., Liu P., Li H. (2019). Phenolic profile, antioxidant activity and anti-proliferative activity of crabapple fruits. Hort. Plant J..

[48] Chen F., Li F., Lu L., Zhang X., Xu X., Li D. (2014). Phenolic profile and changes in the antioxidant activity of crabapple (*Malus domestica* cv Royalty) fruit during maturation on the tree. Int. J. Food Sci. Technol..

[49] Zheng H.Z., Kim Y., Chung S.K. (2012). A profile of physicochemical and antioxidant changes during fruit growth for the utilisation of unripe apples. Food Chem..

[50] Sethi S., Joshi A., Arora B., Bhowmik A., Sharma R.R., Kumar P. (2020). Significance of FRAP, DPPH, and CUPRAC assays for antioxidant activity determination in apple fruit extracts. Eur. Food Res. Technol..

[51] Ma Y., Huang H. (2014). Characterisation and comparison of phenols, flavonoids and isoflavones of soymilk and their correlations with antioxidant activity. Int. J. Food Sci. Technol..

[52] Wang X.H., Zhou S.Y., Qian Z.Z., Zhang H.L., Qiu L.H., Song Z., Zhao J., Wang P., Hao X.S., Wang H.Q. (2013). Evaluation of toxicity and single-dose pharmacokinetics of intravenous ursolic acid liposomes in healthy adult volunteers and patients with advanced solid tumors. Expert Opin. Drug Metab. Toxicol..

[53] Moon S.H., Kim K.T., Lee N.K., Han Y.S., Nah S.Y., Cho S.G., Park Y.S., Paik H.D. (2009). Inhibitory effects of naringenin and its novel derivatives on hyaluronidase. Food Sci. Biotechnol..

[54] Abdullah N.H., Thomas N.F., Sivasothy Y., Lee V.S., Liew S.Y., Noorbatcha I.A., Awang K. (2016). Hyaluronidase inhibitory activity of pentacylic triterpenoids from *Prismatomeris tetrandra* (Roxb.) K. Schum: Isolation, synthesis and qsar study. Int. J. Mol. Sci..

[55] Omar R., Galala A., Badria F. (2019). Multi-target inhibitory activity of some medicinal plants on α-amylase, tyrosinase and hyaluronidase: Potential therapy for treatment diabetes and diabetic complications. ASNH.

[56] Lee E.H., Cho F.B., Kim B.O., Jung H.Y., Lee S.Y., Yoo J., Kang I.K., Cho Y.J. (2020). Functional properties of newly-bred ‘Summer king’ apples. J. Hortic. Sci. Technol..

[57] Piwowarskia J.P., Kissa A.K., Wojciechowskab M.K. (2011). Anti-hyaluronidase and anti-elastase activity screening of tannin-rich plant materials used in traditional Polish medicine for external treatment of diseases with inflammatory background. J. Ethnopharmacol..

[58] Félix-Silva J., Gomes J.A.S., Xavier-Santos J.B., Passos J.G.R., Silva-Junior A.A., Tambourgi D.V., Fernandes-Pedrosa M.F. (2017). Inhibition of local effects induced by *Bothrops erythromelas* snake venom: Assessment of the effectiveness of Brazilian polyvalent bothropic antivenom and aqueous leaf extract of *Jatropha gossypiifolia*. Toxicon.

[59] Shukla S., Gupta S. (2010). Apigenin: A promising molecule for cancer prevention. Pharm. Res..

[60] Stump T., Santee B., Williams L., Heinze C., Kunze R. (2017). The effects of apigenin on cell proliferation and apoptosis in glioblastoma multiforme. J. Pharm. Pharmacol..

[61] Juan M.E., Planas J.M., Ruiz-Gutierrez V., Daniel H., Wenzel U. (2008). Antiproliferative and apoptosis-inducing effects of maslinic and oleanolic acids, two pentacyclic triterpenes from olives, on HT-29 colon cancer cells. Br. J. Nutr..

[62] Bache M., Hein A., Petrenko M., Güttler A., Keßler J., Wichmann H., Kappler M., Emmerich D., Paschke R., Vordermark D. (2019). Evaluation of the betulinic acid–cisplatin conjugate APC and its precursor DE9B for the treatment of human malignant glioma. Chem. Biol. Interact..

[63] Wang J., Li Y., Wang X., Jiang C. (2012). Ursolic acid inhibits proliferation and induces apoptosis in human glioblastoma cell lines U251 by suppressing TGF-b1/miR-21/PDCD4 pathway. Basic Clin. Pharmacol. Toxicol..

[64] Shen S., Zhang Y., Zhang R., Tu X., Gong X. (2014). Ursolic acid induces autophagy in U87MG cells via ROS-dependent endoplasmic reticulum stress. Chem. Biol. Interact..

[65] Tan J., Shen Z.X., Gen W. (2006). Ursolic acid induces apoptosis in colon cancer HT-29 cells. Chin. J. Oncog..

[66] Andersson D., Liu J.J., Nilsson A., Duan R.D. (2003). Ursolic acid inhibits proliferation and stimulates apoptosis in HT29 cells following activation of alkaline sphingomyelinase. Anticancer Res..

[67] Shan J.Z., Xuan Y.Y., Zheng S., Dong Q., Zhang S.Z. (2009). Ursolic acid inhibits proliferation and induces apoptosis of HT-29 colon cancer cells by inhibiting the EGFR/MAPK pathway. J. Zhejiang Univ. Sci. B.

[68] Apak R., Güçlü K., Demirata B., Özyürek M., Çelik S.E., Bektaşoğlu B., Berker K.I., Özyurt D. (2007). Comparative evaluation of various total antioxidant capacity assays applied to phenolic compounds with the CUPRAC assay. Molecules.

[69] Lee F.H., Park H.J., Kim B.O., Choi H.W., Park K.I., Kang I.K., Cho Y.J. (2020). Anti-inflammatory effect of Malus domestica cv. Green ball apple peel extract on raw 264.7 macrophages. Appl. Biol. Chem..

[70] Checker R., Sandur S.K., Sharma D., Patwardhan R.S., Jayakumar S., Kohli V., Sethi G., Aggarwal B.B., Sainis K.B. (2012). Potent anti-inflammatory activity of ursolic acid, a triterpenoid antioxidant, is mediated through suppression of NF-kB, AP-1 and NF-AT. PLoS ONE.

[71] Whatcott C., Han H., Posner R.G., Hostetter G., Von Hoff D.D. (2011). Targeting the tumor microenvironment in cancer: Why hyaluronidase deserves a second look. Cancer Discov..

[72] Liyanaarachchia G.D., Samarasekeraa J.K.R.R., Mahanama K.R.R., Hemalal K.D.P. (2018). Tyrosinase, elastase, hyaluronidase, inhibitory and antioxidant activity of Sri Lankan medicinal plants for novel cosmeceuticals. Ind. Crop. Prod..

[73] Michel P., Owczarek A., Matczak M., Kosno M., Szymanski P., Mikiciuk-Olasik E., Kilanowicz A., Wesołowski W., Olszewska M.A. (2017). Metabolite profiling of eastern teaberry (*Gaultheria procumbens* L.) lipophilic leaf extracts with hyaluronidase and lipoxygenase inhibitory activity. Molecules.

[74] Cargnin S.T., Gnoatto S.B. (2017). Ursolic acid from apple pomace and traditional plants: A valuable triterpenoid with functional properties. Food Chem..

[75] Han N., Bakovic M. (2015). Biologically active triterpenoids and their cardioprotective and anti-inflammatory effects. J. Bioanal. Biomed..

[76] Yadav V.R., Prasad S., Sung B., Kannappan R., Aggarwal B.B. (2010). Targeting inflammatory pathways by triterpenoids for prevention and treatment of cancer. Molecules.

[77] Wozniak L., Skapska S., Marszałek K. (2015). Ursolic acid—A pentacyclic terpenoid with a wide spectrum of pharmacological activities. Molecules.

[78] Lipinski C.A. (2000). Drug-like properties and the causes of poor solubility and poor permeability. J. Pharmacol. Toxicol. Methods.

[79] Gareth T. (2007). Medicinal Chemistry, an Introduction.

[80] Furtado N.A.J.C., Pirson L., Edelberg H., Miranda L.M., Loira-Pastoriza C., Preat V., Larondelle Y., André C.M. (2017). Pentacyclic triterpene bioavailability: An overview of in vitro and in vivo studies. Molecules.

